# Development of Unmanned Excavator Vehicle System for Performing Dangerous Construction Work †

**DOI:** 10.3390/s19224853

**Published:** 2019-11-07

**Authors:** Joosung Lee, Byeol Kim, Dongik Sun, Changsoo Han, Yonghan Ahn

**Affiliations:** 1 Innovative Durable Building and Infrastructure Research Center, Hanyang University, 55 Hanyangdaehak-ro, Sangnok-gu, Ansan-si, Gyeonggi-do 15588, Korea; js4ever@hanyang.ac.kr; 2 Department of Architectural Engineering, Hanyang University, 55 Hanyangdaehak-ro, Sangnok-gu, Ansan-si, Gyeonggi-do 15588, Korea; keemstars@naver.com; 3 Department of Mechatronics Engineering, Hanyang University, 55 Hanyangdaehak-ro, Sangnok-gu, Ansan-si, Gyeonggi-do 15588, Korea; jeniussdi@hanyang.ac.kr; 4 Department of Robot Engineering, Hanyang University, 55 Hanyangdaehak-ro, Sangnok-gu, Ansan-si, Gyeonggi-do 15588, Korea

**Keywords:** unmanned excavator vehicle system, excavator, remote controlled manipulator

## Abstract

Of all the machinery and equipment used on construction sites, excavators are responsible for the greatest number of fatal accidents. Excavation is an inherently risky process due to imprecise work processes and the unstable external environment on most work sites. The resulting accidents cause serious injuries that threaten the lives of not only the excavator pilots but also those working around them. In this study, we propose a mechanical device that is capable of operating the excavator remotely from a nearby safe location such as a site office. To ensure worker safety and at the same time boost the productivity of excavation operations, data from 3D scanners, cameras, and sensors were combined to create a detailed 3D picture of the area surrounding the excavation site to guide a remotely operated excavating system. Rather than modifying the excavator itself, a removable manipulator was mounted on the joystick in the excavator’s cockpit. Our proposed system was tested on a standard commercial excavator to verify its performance for a real-world excavation task.

## 1. Introduction

Excavators, which are primarily used for digging foundations and moving soil on construction sites, are each controlled by an operator and frequently work in close proximity to other workers who are assisting with the excavation or performing other tasks nearby. Space is often limited, and the nature of the excavation task means that there are frequent accidents due to factors such as soil erosion, reclamation, vehicle rollover on unstable surfaces, and even the collapse of structures due to accidents during the construction process [1]. Accidents caused by excavators can not only lead to the failure of the excavator, but also human casualties, either the excavator operator or others working close by. Among the 632 deaths due to machinery and equipment accidents recorded on Korean construction sites from 2009 to 2015, 121 were killed by an excavator, the highest number of fatal accidents caused by any single type of construction machinery or equipment [2]. Common causes of excavator accidents are the deformation of the site due to the excavation work, which by its very nature creates an unstable environment, and excavator rollovers, both of which involve a loss of balance [3,4]. Even under normal excavating conditions, however, changes in irregular and dynamic loads due to soil effects can cause soil erosion, leading to collapses by equipment and materials, structural damage, and excavator accidents [5,6,7,8,9,10,11,12,13,14,15,16,17,18,19,20].

An obvious way to prevent excavator accidents is to utilize unmanned excavators, where the operator is seated safely in a nearby operating center. Earthmoving works using remote control or unmanned excavators bring many benefits in terms of reducing work costs and time, enabling work to proceed safely in hazardous areas where there are serious risks of accidents, collapses and contamination with hazardous material, as well as in areas that would otherwise be inaccessible to human operators. From an economic point of view, technologies for unmanned excavations such as automatic recognition technology for the surrounding environment can speed up the work and simplify the process [21]. Unmanned earthworks equipment can make it possible to carry out much of the construction work that will be required for the National Aeronautics and Space Administration (NASA)’s proposed lunar base before the human occupants arrive [22,23], for example, while another potentially valuable option would be to carry out environmental restoration and relief work in disaster areas and where nuclear power plants have become contaminated with chemicals or radioactive materials [24,25]. However, despite these advantages, research involving unmanned excavators suffers from several limitations that do not reflect the demands of reality. Although a remote disaster recovery excavating system enabling remote operators to perform real-time work in a hazardous environment safely and sustainably was developed and utilized for disaster recovery at the Fukushima plant in Japan [26], the system used required a time-consuming and costly complete overhaul of the excavator to install disaster site-specific inputs, and was also limited by not being able to correct work errors caused by deviations from pre-set working protocols. In 2019, Doosan Infracore of South Korea developed a Tele-operation system to remotely operate construction equipment and machinery. Based on a 5G communication network, the company claims that it can be controlled remotely from anywhere in the world, with a very fast response speed of 20 msec. However, this technology can only be applied to specific models developed by their company, and it would thus be difficult to utilize for commercial excavators from other manufacturers, which have many different types of cockpit and joysticks.

Research on autonomous driving has been another important field of research related to automated excavators. Zweiri [27] compared the performance achieved by parameter analysis methods such as the Newton-Raphson, generalized Newton and least squares methods for parameter optimization of a dynamic model of the arm of an unmanned excavator. However, the unmanned excavator analyzed in Zweiri’s paper suffered from several limitations, as not only was the cost of converting the excavator for unmanned operation high, but it would also be hard to commercialize because of the poor efficiency achieved in the field trials. Bender et al. [28] presented a predictive operator modeling technique for virtual prototyping of a hydraulic excavator and proposed a methodology for simulating the degrees of freedom of the boom, arm, and bucket. Although they suggested an alternative simulation method for the optimization of excavation work, they failed to provide details of the methodology utilized or the results of field tests of unmanned excavators. Zhou et al. [29] implemented a linkage algorithm from the boom cylinder to the bucket cylinder to optimize excavator driving and save energy; they also proposed a new prediction-based stochastic dynamic programming control methodology. Although this provides useful information for optimizing the excavator control process, it is of only limited utility when it comes to unmanned excavator operations. Kim et al. [30] proposed a vision-based activity identification methodology for identifying the dump and excavator status for unmanned excavation. Unfortunately, their vision-based method was less reliable than recent technologies such as deep-learning based on real-time 3D scan data, and they identified significant differences in the results depending on the analysis frequency. Kim et al. [31] put forward the concept of a robotic excavator, utilizing a pre-defined work algorithm to guide an automated excavator. They encountered difficulties in applying real-time information collection methods such as 3D Scan and Deep Learning to cope with the varied conditions and events encountered in the field, however, and there was a distance beyond which operations became impractical. These optimization and unmanned excavator studies developed a wide variety of methods to calculate the optimal arm and boom drive algorithm, albeit with the common limitation that either the excavator or its arm must be fully remodeled, making it difficult to accurately reflect rapidly changing field conditions in real time. These drawbacks make it unfeasible to commercialize any of these proposed systems, as even if such a product were to be brought to market, its utility would be limited and the price uneconomically high.

To build on the existing research in this area, the next step is to study remote excavator manipulation methods based on remote control systems. An unmanned control system based on a removable manipulator should be both effective and easy-to-use across a relatively wide range of environmental conditions. A remote control system is expected to be capable of achieving the same performance as a conventional excavator because it collects real-time images from multiple sensors and delivers these to the operator, who is located in a nearby safe indoor environment. Because the operator is not physically present in the cab, he or she is not at risk in the event of a large-scale accident occurring when excavating groundworks, underground frame construction, or demolition, all of which are difficult environments to work in, with a high risk of burial, rockfall or collapse. The detachable manipulators developed for such projects can be readily adapted and installed on a variety of equipment by simply modifying the manipulation method used for the control stick. In early work along these lines, Lee et al. discussed the conceptual process and reported the results for the development and implementation of a prototype unmanned excavation system [32]. Taking this further, in this paper, we report the development of an innovative system whereby a manipulator is installed in the cockpit of a commercial excavator to enable remote operation from a safe nearby location, including an appropriate communication method between the remote control station and manipulator, and the associated remote control system.

## 2. Research Methodology

### 2.1. Scope of Research

As part of the development of an unmanned excavation system for use on real-world construction work sites, the study reported in this paper developed (1) appropriate remote control stations, (2) an innovative manipulator for unmanned operation of excavators, (3) communication networks linking the station and excavator, and (4) a new sensor system for the measurement of excavator attitude. The subsequent performance analysis verified the mechanical performance of the proposed system.

### 2.2. Research Methodology

**Figure 1:**
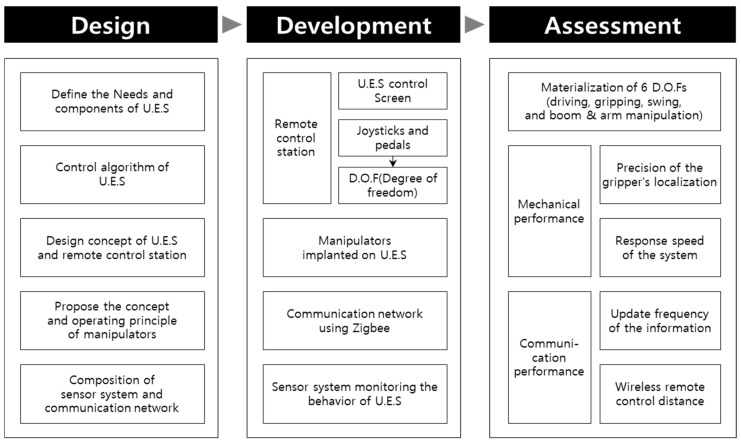
Research methodology.

The research method adopted for the development of the unmanned excavation system (U.E.S.) and its individual components is shown in Figure 1 below.

To develop an unmanned excavation system that delivers the same work performance as existing conventional excavators, the procedure listed below was adopted:

(1) Define the needs and performance goals for an unmanned excavating vehicle system;
(2) Design the manipulation algorithm, system concept and its components; and
(3) Develop the system structure and individual components, including the remote control station (joysticks and cockpit), the excavator’s manipulator, the communication network environment, and the behavior control sensor.

To analyze the technical performance of the unmanned excavation system, five types of performance analysis in 3 main categories were utilized.

(1) Materialization of 6 D.O.Fs: driving modes, gripping modes, swing modes, and boom and arm manipulation modes;
(2) Mechanical performance: (a) necessary gripper position measurement accuracy and (b) optimum remote control response speed; and
(3) Communication performance: (a) information update period required by the excavator, and (b) the desired behavior and optimum distance for the remote wireless communication.

## 3. Design of Unmanned Excavating Vehicle System

### 3.1. Control Algorithm to Operate the Gripper and Behavior of the Unmanned Excavation Vehicle System

**Figure 2:**
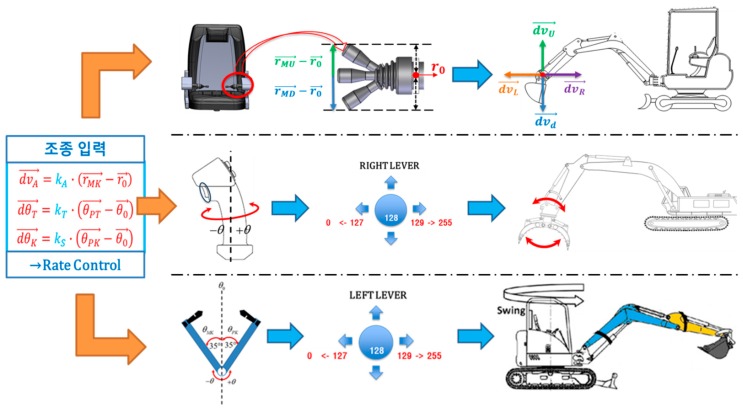
Excavator control algorithm using manipulator.

The operation of excavators from remote control stations is inevitably more difficult than manned operations due to the need to address problems such as ground collapse, falling rocks, and so on. It is therefore vital to ensure that the unmanned control methods are intuitive and have similar operation methods to those of the actual excavators. The operating algorithm implemented for the cockpit and manipulation joystick is shown in Figure 2.

The input signals that control the manipulator installed on the unmanned excavator are divided into upper and lower signals based on a default value of 128 bits. These signals operate individual tasks such as forward and backward, up and down, and right and left for driving and turning, as well as operations such as gripping, swing, boom and arm manipulation. In particular, the control displacement of the joysticks is set to equal the actual range of posture control and operation displacement of the gripper, arm and body of the real-world excavator.

### 3.2. Remote Control Station to Control the Manipulator

**Figure 3:**
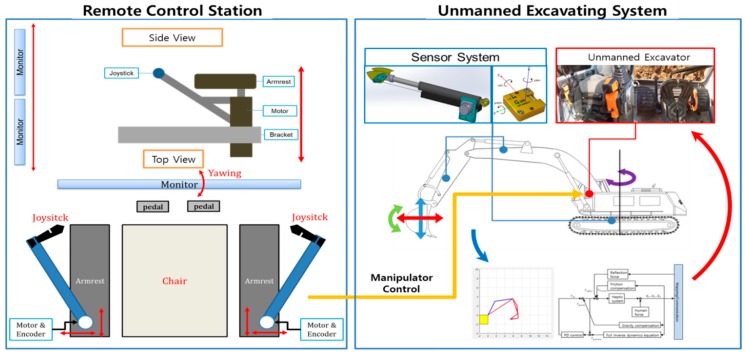
Concept of unmanned excavating system.

The unmanned excavation system developed for this project consists of three main components: (1) the unmanned excavator control system in remote space, (2) the unmanned excavator for on-site work, and (3) the sensor responsible for providing excavator attitude control and position information. The unmanned control components consist of a control unit that remotely operates the levers and pedals inside the excavator cabin, a positioning GPS, and a bucket attitude control sensor. Real-time visualization is possible because the videographic data collected by the IP camera installed on the roof of the excavator is transferred to the PC in the workspace through a commercial RF communication module. The operator, who is located in a nearby building, manipulates the remote control equipment to control the excavator on the work site. Figure 3 shows the underlying principles of the unmanned excavation system and the associated remote control system.

### 3.3. Manipulator Implanted on the Excavator Controllers

In this proof-of-concept study, the remote control station drives the manipulator attached to the excavator’s conventional controls via a Zigbee communication link. The manipulator consists of four control units, each of which manipulates one of the two levers and two pedals that constitute the original joysticks of the excavator. The manipulator’s four control units manipulate the joystick based on the command signals received by the main controller. The use of this type of remote control system enables unmanned excavators to be driven with accuracy and safety, just as they would be if manned.

**Figure 4:**
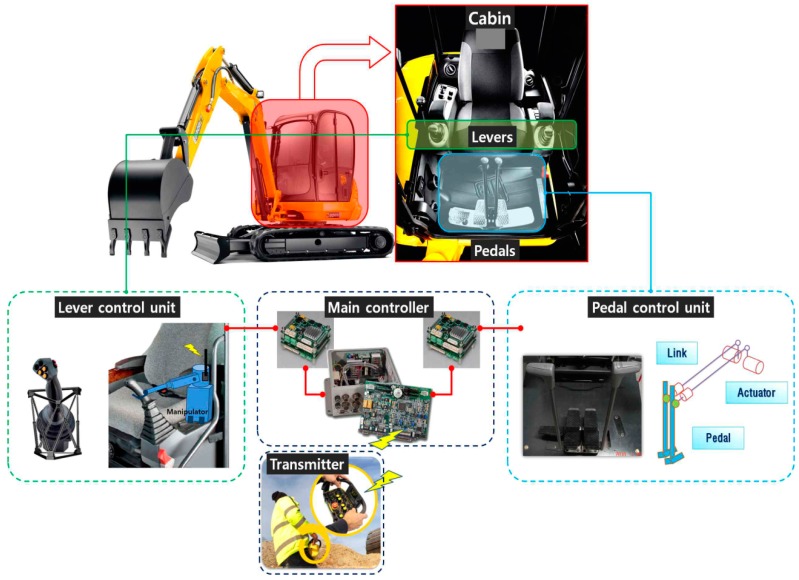
Manipulation system installed on the excavator.

The manipulator components installed on the control boom, the bucket, and the stick in the machine’s cabin receive operational data from the two levers of the remote control station, which are converted to an 8-bit signal and sent to the manipulator via the Zigbee communication protocol. This then drives the excavator joystick to the left, right, top and bottom based on the signals received. The operating principle for the link between the joystick and the manipulator mounted on the excavator is shown in Figure 4. The input signal from the remote control transmitted to the main controller of the excavator installed on the joystick takes the form of an 8-bit electric signal, causing the main controller to operate the excavator by manipulating the lever control unit and pedal control unit in response to the remote operator’s instructions.

**Figure 5:**
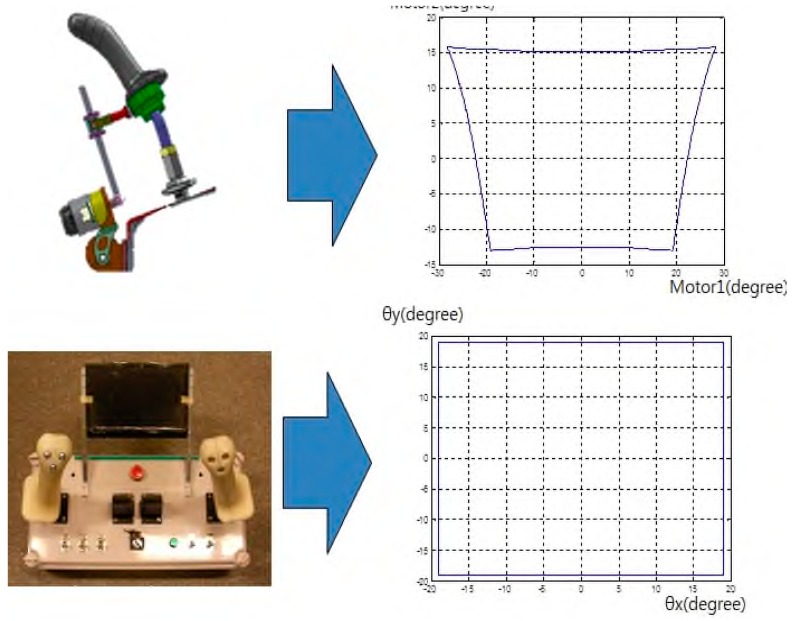
Workspace area: difference between the remote controller and manipulator.

Here, the work space form of the remote control is different in size and shape from that of the manipulator. The lever-operated manipulator has a trapezoidal shape due to the slide joint operation of the lever, but the joystick workspace of the remote control is a perfect rectangle, as shown in Figure 5. This was dealt with by applying a coordinate shape-mapping procedure to the control workspace between the two.

A total of six points, namely, the four vertices at the boundary of the manipulator workspace and the left and right end points at the center, are estimated using data from the encoder. The linear equation between each point can then be derived based on the contrast between the coordinate data and the joystick of the remote controlled excavator, and matched to the boundaries of the work space.

### 3.4. Sensor System to Monitor the Localization and Attitude of the Excavator

Based on the command signal value, the excavator classifies the signals as either a micro mode for driving in the task space or a macro mode for driving the cylinder in the joint region. In the macro mode, the actuator is directly driven by visual feedback, but in the micro mode, the excavator decides whether to perform the task in accordance with the position and speed regime fed back through the sensor and kinematic workspace. A potentiometer and encoder type stroke sensor, a Novatel GPS position sensor, and a tilt sensor are also used to collect necessary information such as the excavator’s attitude and gripper position.

### 3.5. Communication Network between the Remote Control Station and Manipulator

A wireless communication system between the control station and manipulator implanted in the excavator is incorporated to remotely control the excavator. Zigbee transceiver modules are attached to both the remote station and the excavator, enabling the station to be configured to transmit driving and arm operation commands to the appropriate manipulator components. The RF communication commercial module, which has an 80 MHz bandwidth and a 5 GHz frequency domain, transmits video information from the IP camera to the remote operator.

## 4. Implementation of Unmanned Excavating Vehicle System

### 4.1. Remote Control Station to Control the Manipulator

To ensure the same performance is achieved as that delivered by excavators with human operators, a new remote control algorithm was developed for navigation, gripper operation and body swing. The new system supports 6 D.O.F (degrees of freedom) for the remote control joystick manipulation device, representing four driving modes (forward, backward, left-turn, right-turn), two gripping modes (grasp and roll up, open and roll down), two swing modes (left-swing and right-swing), and four boom and arm manipulation actions (forward, backward, up and down).

**Figure 6:**
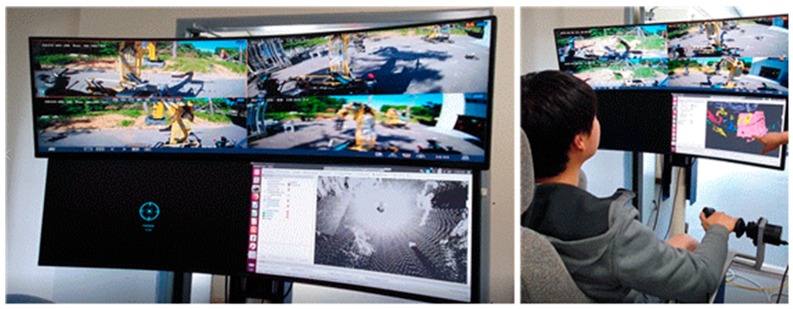
Implementation of remote control station: screen of r.c station (**left**), real-time operation using remote control station and screen (**right**).

The new remote control station developed to implement the excavator’s degrees of freedom, shown in Figure 6, consists of an integrated controller composed of control arms, control pedals, a monitoring system, and a camera system. The screen in front of the controller’s seat visualizes the entire field of view captured by cameras attached above the driver’s position on the roof of the cockpit in real time, along with the 3D scan data transmitted by the mobile 3D scan robot.

### 4.2. Unmanned Excavator Controlled by Remote Control System and Manipulator

**Figure 7:**
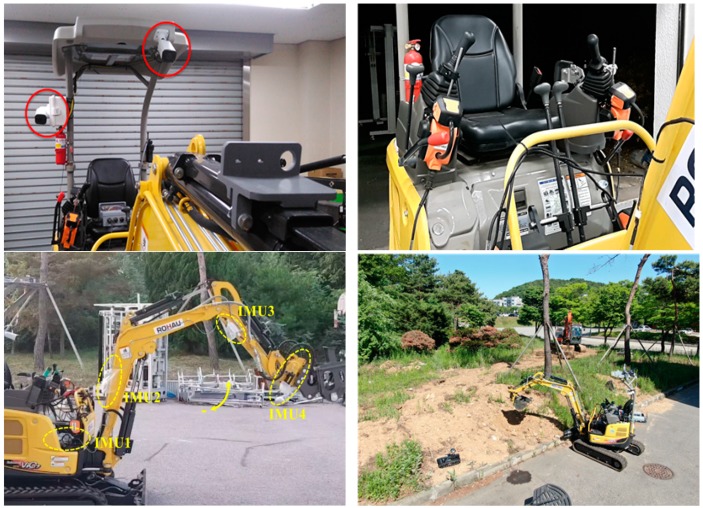
Implementation of unmanned excavator: locations of the two cameras providing information to the operator(**top-left**), cockpit with manipulator(**top-right**), sensor system on boom (**bottom-left**), and excavation work performed by remote control (**bottom-right**).

As described earlier, the manipulator attached to the excavator operates the lever based on the 8-bit control signal it receives from the remote control station. Figure 7 shows the positions of the cameras (top left) and the manipulator installation in the cab of the excavator (top right). RTK GPS is utilized to measure the center position and swing angle of the main body of the excavator, and an IMU sensor based on the Micro Electro Mechanical System (MEMS) is attached to measure the angular displacement of each joint and cylinder displacement. These two sensors measure the overall position and attitude of the excavator. Based on the position data and the angle data measured by the GPS and 4 IMU sensors, respectively, the position of the excavator bucket is calculated using Forward Kinematics. The center position of the main body, the angle of each joint, and the location data of the bucket stand are transmitted to the remote control station, providing the operator with information on the current position and attitude of the excavator.

The working prototype of the unmanned excavator developed for this project is shown in the bottom part of Figure 7.

## 5. Analysis of the Performance of the Proposed System

### 5.1. Comparison of Target and Real-World Performance Achieved by the Proposed System

A set of target performance indicators were established during the initial stage of development for unmanned excavators. These were: (1) the implementation of optimal D.O.F. for performing actual excavation work, (2) the selection of precise gripper positions for remote operation, (3) an adequate response speed of the excavator in response to remote control signals, (4) the optimization of the excavator attitude information update frequency for real-time safety management of unmanned excavating tasks, and (5) a greater maximum distance for the wireless remote control.

All these indicators represent basic performance standards that enable actual operations to be carried out by an unmanned excavation system; these targets were established based on the existing highest performance indicators achieved using the same (Zigbee) communications system. The performance achieved are compared with each target performance indicator are shown in Table 1.

The main purpose of this research was to develop an unmanned excavator system and a remote controlled manipulator to satisfy the primary mechanical performance, namely the D.O.F necessary for each excavator action, a correct operational response to the control signal, and the precision of the localization. In particular, the targets for communication performance, particularly the response speed of the system and the maximum distance for the remote control, were not high-priority aspects of the technical challenge addressed here; future work that incorporates a 5G application is planned to improve communication performance once the initial system development is complete. In the development stage of the unmanned excavation system, the use of a communication network through the Zigbee module alone was deemed sufficient to verify performance. Accordingly, the target of response speed and remote wireless control distance set in Table 1 will be achieved at the Future work, which will be equipped with 5G communication module of LG U Plus. In more detail, 30 msec for response speed is a combination of 20 msec for sending and receiving data between communication modules using 5G networks and 10 msec for analyzing and processing control signals in the control panel of manipulator. In addition, the wireless remote control distance can be close to infinity, because 5G communication modules can be used for remote control using networks distributed around the world.

The degree of freedom factor achieved 6 D.O.F., sufficient to cover all the operational parameters required for commercial excavators. The precision of the gripper’s localization was ±1.8 cm, which is better than the previous best of ±3 cm. The response speed of the excavator to remote control was recorded at 357 msec, and the response speed between the wireless modules was 50 msec. The wireless remote control distance was 10.25 m. Further details are provided below.

### 5.2. Materialization of 6 D.O.Fs

A test was performed to verify the operational performance of the unmanned excavator under the control of the remote control. To determine whether the unmanned excavator was capable of the same level of excavating work as the manned excavator, the implementation status of 6 D.O.Fs, consisting of two driving modes (forward, backward, left-turn, right-turn), one gripping mode (grasp and roll up, open and roll down), one swing mode (left-swing and right-swing), and two boom and arm manipulations (forward, backward, up and down), was measured. Also to determine whether the D.O.F were all actually implemented, the number of unmanned excavator joints successfully driven by the joystick of the remote control station was visually assessed.

**Figure 8:**
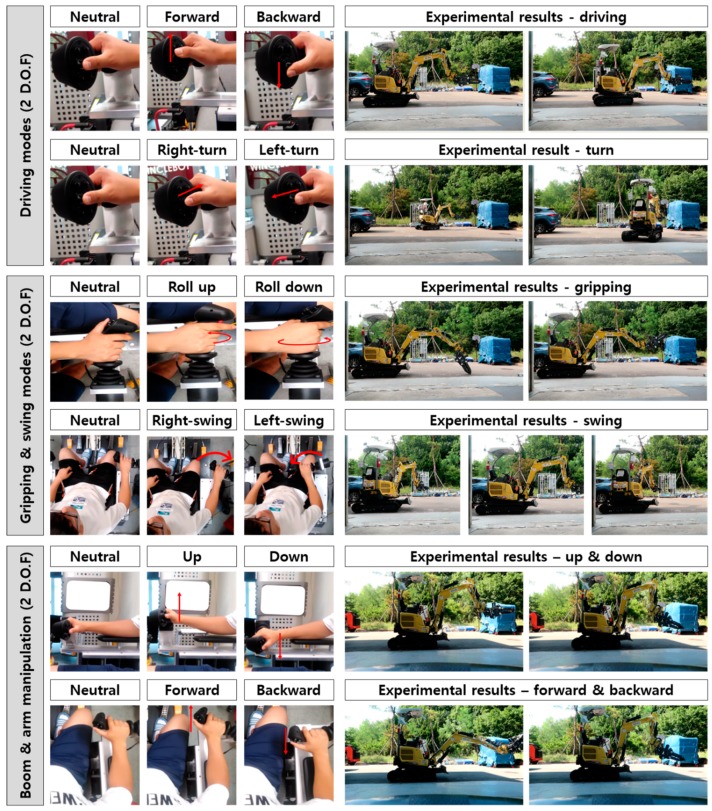
Excavator operation test based on 6 D.O.Fs.

Figure 8 shows the experimental results for the driving modes, gripping modes, swing modes, and boom and arm management. As shown in the figure, the operation was successful for all 6 D.O.Fs targeted in the design phase, and it was deemed possible to control unmanned excavator operations across the same range as that achieved by manned operation.

### 5.3. Mechanical Performance of the Proposed System

To verify the mechanical performance of the unmanned excavation system, the precision of the gripper’s localization and the speed of the system’s response to commands issued by the remote control were analyzed.

#### 5.3.1. Precision of the Grippers’ Localization

**Figure 9:**
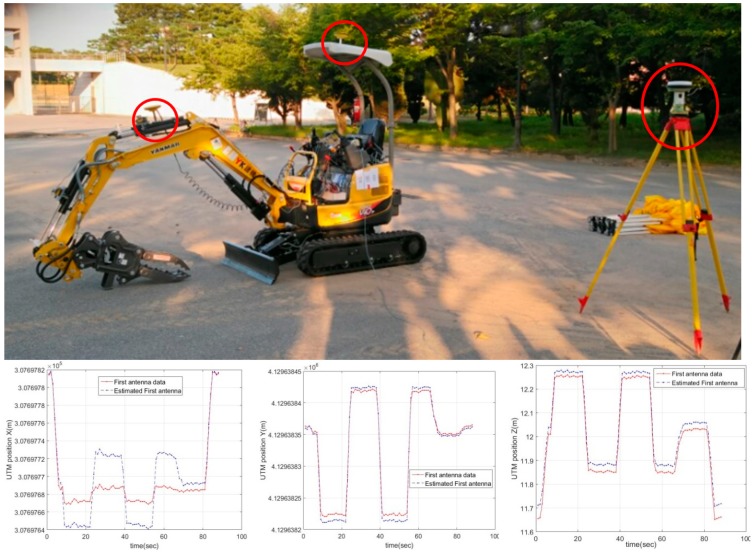
Precision of gripper localization.

First, in order to analyze the precision of the gripper’s localization, the X, Y, Z coordinates of the gripper were measured using a CAN Analyzer in real time. The position of the gripper was then calculated relative to the position of the second GPS antenna, which was estimated and calculated from the reference coordinates at which the GPS antenna was installed. This was repeated 5 times and the mean value for the five measurements found to be ±1.8 cm, confirming that a stable and repeatable performance had indeed been secured. Figure 9 shows the test environment and the results.

#### 5.3.2. Response Speed of the System

To assess the response speed of the system when sent a command by the remote control, the difference between the remote control input time (measured by the CAN Analyzer) and the actual response time (measured by RTK-GPS and the CAN Analyzer) were measured. Once the RTK-GPS and Can analyzer had been set up and calibrated, an open environment that would not interfere with communications, containing no ceilings or walls, was established. The unmanned excavator was then manipulated at a distance of no more than 10 m from the remote control station.

**Figure 10:**
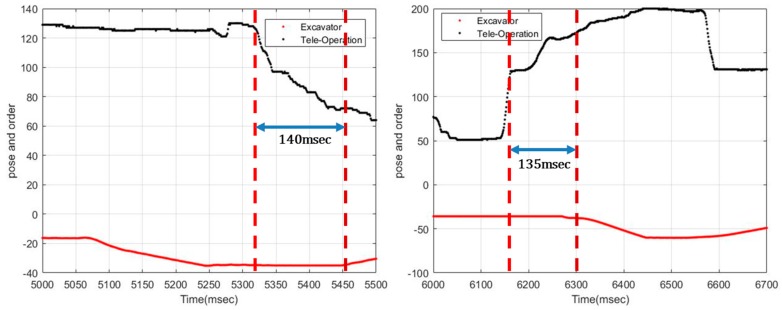
Two sample results for the response speed of the excavator.

Since the results of each measurement were different, the average of their response time was obtained by conducting five tests. Figure 10 shows two of the five results. The overall average was found to be 0.357 s, confirming that there was no significant work response delay affecting the performance of the proposed unmanned excavation system. This reading of 0.357 msec represents the “total time” taken for the actual excavator drive to respond, and includes 50 msec for the actual intercommunication process, 100~200 msec for the latency of the camera, and 150 msec for the hysteresis of the hydraulic system. This indicates that our proposed system actually has a response speed of about 50 msec.

### 5.4. Communication Performance of Proposed System

Speedy and accurate communication between the excavator and the control station is vital, and the performance of the communication channel has a direct impact on the precision and productivity of excavation work. We therefore verified the communication performance by measuring the update frequency of the attitude information and the maximum acceptable distance between the operator and the unmanned vehicle for adequate wireless telecommunication.

#### 5.4.1. Update Frequency of the Attitude Information

**Figure 11:**
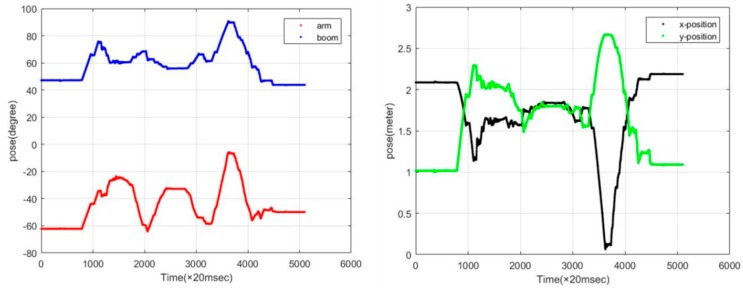

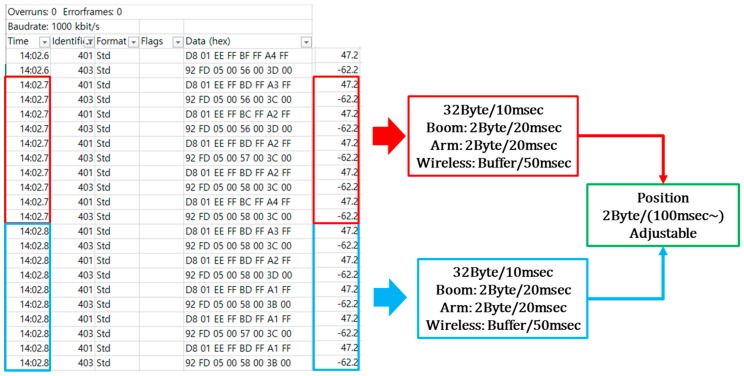
Update frequency of the attitude information.

Updating the attitude information proceeds via 3 steps: (1) integrate the action information for the boom, arm and gripper, as measured by sensors; (2) calibrate their positions and angles; and (3) transmit the precise position values for both the excavator and gripper to the control system using UART and CAN communication devices. The data presented in Figure 11 confirm that performance was achieved when the attitude value was transmitted and received every 200 msec (5 Hz).

#### 5.4.2. Wireless Remote Control Distance

**Figure 12:**
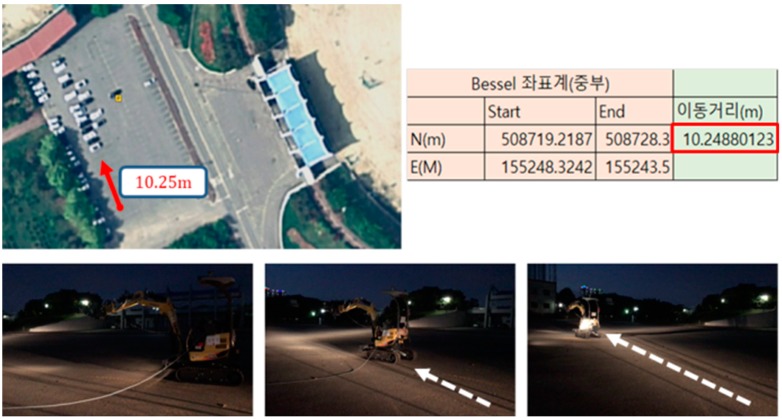
Maximum acceptable distance between the operator and the unmanned vehicle.

For the wireless remote control distance analysis, RTK-GPS units were mounted on both the remote control station and the excavator. The results showed that remote control was no longer possible when the excavator was at a distance of 10.25 m or more from the remote operator. Figure 12 shows the test results for the maximum distance between operator and the unmanned excavation system.

## 6. Conclusions

The findings of this study suggest that an unmanned excavation system can indeed be designed that overcomes the limitations of previous unmanned excavator research, ameliorating many of the hazards associated with operating an excavator on a construction site and preventing accidents. The technical performance of our proposed system was verified by an analysis of its mechanical and communication performance.

However, the fact that the proposed unmanned excavating system is operated by the Zigbee communication network is a major limitation of this study. By utilizing the Zigbee communication module, the system was successfully operated with no problems in an open space with a radius of 50 m centered on the remote control station. However, if obstacles exist between the communication modules, latency of more than 50 msec occurred, seriously degrading the response speed. In addition, the response speed of the excavator dropped considerably as the distance between the control station and the excavator increased. Moreover, if the temperature of the module were to increase due to system overload or prolonged driving, communication performance would also be degraded.

To overcome this problem, an unmanned excavating system with 5G telecommunication network connection is currently being developed. Utilizing a 5G network is expected to dramatically improve both the response speed and wireless remote control maximum distance. In particular, the same communication module will be equipped with our unmanned excavation system, just as “Tele Operation of Doosan Infracore”, which has the world’s best communication performance for unmanned excavators, is equipped with 5G communication module of LG U Plus. Based on 5G communication module, remote control distance and response speed of unmanned Excavation System will be greatly improved.

Our proposed unmanned excavation system can be used not only for high-risk tasks such as earthworks and demolition, but also to perform risky recovery operations in disaster areas or places where it is not safe for people to go, such as inside high radiation zones in nuclear facilities. Installing manipulators in an excavator is just one possible application for this new approach, which has the potential to become a core technology for unmanned heavy equipment by providing a versatile way to modify a wide range of equipment to accommodate remote operation. A detailed study of the operation principle of the manipulator and the gripper manipulation methodology is currently underway. 

## Figures and Tables

**Table 1:** Target performance and achievement of proposed system.

Performance Indicator of U.E.S.	Unit	World’s Highest Performance	Target	Actual	Measurement Methods
D.O.F of remote control joysticks	DOF	8DOF (Japan/Hitachi, ASTACO)	6	6	Visual checks of the number of joints operated by remote control
Precision of the grippers’ localization	cm	±3 cm (Japan/Komatsu, PC210LCi-10)	±5 cm	±1.8 cm	Average of 5 measurements
Response speed of the system	Sec	0.020 s (South Korea/Doosan Infracore, Tele-operation)	0.03	0.357 s	Average of 5 measurements
Update frequency of the attitude information	Hz	5 (U.S/CMU)	3 Hz	5 Hz	Calculated based on the average update frequency achieved for the attitude data
Wireless remote control distance	m	50 (Germany/Siemens)	∞	10.25	Maximum distance for effective signal transmission between the RTK-GPS sensors installed in the excavator and remote controller
